# Optical Coherence Tomography Angiography Changes in Diabetic Macular Ischemia after Systemic Normobaric Oxygen Therapy

**DOI:** 10.18502/jovr.v20.15354

**Published:** 2025-05-19

**Authors:** Diba Idani, Seyed Mohammadjavad Mashhadi, Hamze Babaei, Farideh Sharifipour, Ramin Nourinia, Bahareh Kheiri, Arezoo Miraftabi

**Affiliations:** ^1^School of Medicine, Shahid Beheshti University of Medical Sciences, Tehran, Iran; ^2^Ophthalmic Research Center, Research Institute for Ophthalmology and Vision Science, Shahid Beheshti University of Medical Sciences, Tehran, Iran; ^3^Department of Ophthalmology, Labbafinejad Medical Center, Shahid Beheshti University of Medical Sciences, Tehran, Iran; ^4^Department of Epidemiology and Biostatistics, Schulich School of Medicine & Dentistry, Western University, Ontario, Canada; ^5^Eye Research Center, Five Senses Health Institute, Moheb Kowsar Hospital, School of Medicine, Iran University of Medical Sciences, Tehran, Iran

**Keywords:** Diabetic Macular Ischemia, Foveal Avascular Zone, Optical Coherence Tomography Angiography, Oxygen Therapy

## Abstract

**Purpose:**

To evaluate vascular changes on optical coherence tomography angiography (OCTA) in patients with diabetic macular ischemia (DMI) after systemic normobaric oxygen (NBO) therapy.

**Methods:**

This before–after interventional studyincluded 26 eyes of 26 patients with DMI.Macular OCTA was performed before and after 1 hour of 100% NBO therapy at a flow of 10 L/min delivered by face mask. As primary outcomes, changes in OCTA metrics were evaluated using the paired *t*-test. Subgroup analyses were performed based on gender. The secondary outcomes included identifying parameters correlated with best-corrected visual acuity (BCVA) and factors associated with improvement in OCTA parameters.

**Results:**

The patients included 15 males and 11 females aged 59.48 ± 9.67 years. Overall, no significant change was observed in retinal thickness; however, there was a significant decrease in retinal thickness among females and a significant increase among males (*P* < 0.001). The foveal avascular zone (FAZ) decreased significantly from 0.38 ± 0.14 to 0.34 ± 0.12 mm^2^ (*P = *0.035). Superficial capillary plexus vessel density (SCP-VD) and deep capillary plexus vessel density (DCP-VD) at fovea increased from 13.5 ± 6.37 to 14.98 ± 6.33% (*P = *0.059) and from 24.61 ± 6.75 to 26.59 ± 6.16% (*P = *0.022), respectively. In males, BCVA correlated significantly with baseline DCP parameters but corresponded with none of the SCP parameters. In females, BCVA significantly correlated with pre-O2 DCP-VD of the perifoveal inferior quadrant. Finally, regression analysis did not show any parameter that could predict a favorable response.

**Conclusion:**

Using OCTA, we observed a decrease in FAZ and an increase in DCP-VD at fovea after short-term NBO therapy for patients with DMI.

##  INTRODUCTION

Diabetes mellitus (DM) is a leading cause of vision loss due to diabetic maculopathy and proliferative diabetic retinopathy (PDR). Diabetic maculopathy includes diabetic macular edema (DME) and diabetic macular ischemia (DMI) or a combination thereof. DMI, a vision-threatening irreversible complication of DM, is characterized by capillary occlusion and dropout in the macula with enlargement and/or irregularity of the foveal avascular zone (FAZ). The presence of DMI is a biomarker of poor vision in patients with diabetes^[1,2,3]^ and adversely affects the visual outcomes of intravitreal injections of anti-vascular endothelial growth factors (VEGFs) for DME.^[4]^ Progressive FAZ enlargement occurs at a rate of 5–10% of baseline FAZ area per year in eyes with established macular ischemia and could be considered a predictor of vision loss.^[5]^

DMI is diagnosed by fluorescein angiography (FA) or, more recently, optical coherence tomography angiography (OCTA). The Early Treatment Diabetic Retinopathy Study (ETDRS) has classified DMI based on capillary loss, FAZ size, and FAZ outline compared to reference photographs.^[6]^ However, currently, there is no widely accepted OCTA-based classification for DMI and no established normative databases for OCTA metrics.

OCTA is a noninvasive technique that can visualize the retinal capillary plexus and outlines of FAZ without the need for intravenous dye injection. Another advantage of this method over FA is that the volumetric angiographic information of superficial capillary plexus (SCP) and deep capillary plexus (DCP) could be acquired separately. Before OCTA, the presence of DMI could have been missed, both with and without FA. However, with the advent of OCTA, DMI is detected more frequently, either alone or concomitant with DME. DMI has been reported in 29.4% of cases with clinically significant macular edema (CSME) and 77.2% of eyes with PDR, and it remains an important cause of vision loss in these patients.^[1]^

While DME can be treated by intravitreal injection of anti-VEGF agents or macular photocoagulation (MPC),^[7]^ no approved treatment for DMI exists.^[8,9]^ Several studies have reported the beneficial effects of systemic normobaric oxygen (NBO) therapy on visual function (visual acuity, electroretinography) and retinal structure (OCT, FA) in patients with diabetes.^[10,11,12]^ Moreover, previous studies on NBO therapy in patients with DMI have observed promising results.^[13,14]^

In this study, we aimed to evaluate macular vascular changes in OCTA in patients with DMI after 1 hour of systemic NBO therapy.

##  METHODS

This before–after study was conducted at the Department of Ophthalmology, Labbafinejad Medical Center, Tehran, Iran. The study protocol adhered to the Tenets of the Declaration of Helsinki and was approved by the Ethics Committee at the Ophthalmic Research Center, Shahid Beheshti University of Medical Sciences. Informed consent was obtained from all participants.

Patients with a best-corrected visual acuity (BCVA) of < 20/30 and DMI identified on OCTA scans were enrolled in the study. Based on the ETDRS-DMI classification, patients had grades 2 to 4 DMI. ETDRS classification is based on capillary loss, FAZ size, and FAZ outline. Accordingly, grade 2 is defined as definite capillary loss (D), FAZ size 300–500 µ, with < 1/2 FAZ outline destroyed. Grade 3 describes moderate capillary loss (M), FAZ size > 500 µ, and ≥ 1/2 FAZ outline destroyed. Grade 4 refers to severe capillary loss (S), with FAZ outline completely destroyed.^[6]^ All patients underwent a complete ocular examination including BCVA, slit-lamp examination, tonometry using a calibrated Goldmann applanation tonometer, and dilated fundus examination.

The exclusion criteria were as follows: presence of active PDR and center-involved DME (CI-DME) with central subfield thickness of > 300 µm, presence of intraretinal cyst on multilayer macular scans, any retinal disease other than diabetic retinopathy, significant media opacities, history of glaucoma, intraocular surgery including intravitreal anti-VEGF injection, cataract surgery or laser photocoagulation within the past three months, presence of vitreomacular traction, severe chronic obstructive pulmonary disease or any contraindication for oxygen therapy, anemia, uncontrolled hypertension or diabetes, and renal failure. For each patient, baseline OCTA was obtained, followed by administration of 100% oxygen by a face mask at a flow rate of 10 L/min for 1 hour. OCTA was repeated immediately after oxygen therapy. Oxygen saturation was measured before and after NBO therapy using a pulse-oximeter (Heart Sure A320 Pulse Oximeter).

OCTA images were acquired by the Avanti RTVue XR (Optovue, Inc.) software version 2018,1,0,43. OCTA scans were captured in both eyes in 6 × 6 volume cubes by the same experienced operator. The data from SCP, DCP, and full-thickness retina were used for analysis. OCTA scans were thoroughly evaluated by two authors (RN, FS). We excluded scans with a quality of < 5/10, signal strength index (SSI) < 35, and /or artefacts such as segmentation errors, motion artifacts, blinking, or double vessel pattern. The device obtains 304 × 304 volumetric A-scans at a rate of 70,000 A-scans per second, in approximately 3 seconds. Full-thickness retinal scans are automatically segmented into the superficial and deep inner retinal vascular plexuses, outer retina, and choriocapillaris. The superficial retinal slab on this device extends from 3 µm below the internal limiting membrane (ILM) to 15 µm below the inner plexiform layer (IPL). The deep retinal slab extends from 16 μ m below the IPL to 69 μ m below the IPL. The superficial vascular plexus shows the vasculature in the retinal nerve fiber layer (RNFL) and ganglion cell layer (GCL). In contrast, the deep vascular plexus shows the vascular plexuses from the IPL and inner nuclear layer (INL) junction to the INL and outer plexiform layer (OPL) junction.

We recorded OCTA-derived vessel density (VD) in the fovea (1 × 1 mm), parafovea (central 3 × 3 mm), and perifovea (6 × 6 mm) areas and subdivisions of each region (including superior and inferior hemiretinas, and superior, inferior, nasal, and temporal quadrants) for both SCP and DCP. Retinal thickness was also obtained for the regions mentioned above. Additionally, FAZ parameters including area, perimeter, and acircularity index were recorded. The acircularity index is the ratio of the measured FAZ perimeter to the perimeter of a circle with the same area.^[15]^ The FAZ area was redrawn if the automated tracing was erroneous. All measurements were obtained before and after oxygen therapy.

### Statistical Analysis

Based on a previous study,^[13]^ a sample size of 26 eyes was calculated. However, we enrolled 30 patients to compensate for possible low-quality OCTA images. Statistical analyses were performed using SPSS (IBM SPSS Statistics for Windows, Version 22.0). Data were presented in means ± SD and number. Paired *t*-test was used to compare values before and after oxygen therapy. Considering different FAZ areas between males and females,^[16,17,18]^ we assumed that gender might affect the patients' baseline parameters. Therefore, all analyses were compared between males and females. Appropriate correlations were sought for each parameter, and the percentage of eyes showing favorable responses was determined. Favorable responses were defined as an increase in vessel density, a decrease in FAZ size or perimeter, and a decrease in retinal thickness. Regression analysis was used to assess parameters associated with improvement. *P*-value < 0.05% was considered statistically significant.

##  RESULTS

Overall, 30 consecutive patients with DMI grades 2–4 were enrolled. After discarding the OCTA images with low quality or artefacts, the data from 26 eyes of 26 patients including 15 males and 11 females were used for the analysis. The patients' mean age was 59.48 ± 9.67 (range, 44–77) years. Sixteen eyes were pseudophakic; 25 eyes had received intravitreal bevacizumab (IVB) with the last session being more than three months prior to the study; 23 eyes had received panretinal photocoagulation; 23 eyes were at the stage of regressed PDR; and 3 eyes showed the signs of severe non-PDR. BCVA was 0.41 ± 0.27 logMAR and intraocular pressure was 13.26 ± 3.17 mmHg with a cup/disc ratio of 0.35 ± 0.15. No adverse effect was observed secondary to oxygen therapy.

Oxygen saturation increased significantly after NBO therapy (*P* < 0.001) [Table 1]. There was no significant difference between the quality of OCTA images pre- and post-oxygen therapy. No significant change occurred in retinal thicknesses in the foveal, parafoveal, or perifoveal regions. There was a significant decrease in the FAZ area (*P = *0.035), but no significant change was observed in the perimeter and acircularity index [Table 1].

### SCP VD Changes After O ${}_{2}$ Therapy

SCP VD at fovea increased from 13.5 ± 6.37% to 14.98 ± 6.33%, although it did not reach the significance level (*P = *0.059). SCP-VD at the temporal parafoveal area increased from 38.67 ± 4.88 to 40.56 ± 6.16 (*P = *0.052) [Table 1].

### DCP VD Changes After O ${}_{2}$ Therapy

Among the DCP parameters, foveal VD increased from 24.61 ± 6.75% to 26.59 ± 6.16% (*P = *0.022). No other significant change was observed in the DCP-VD [Table 1].

**Table 1:** Changes in OCTA parameters after 1 hour of normobaric oxygen therapy

	**Before oxygen therapy (Mean ± SD)**	**After oxygen therapy (Mean ± SD)**	***P*** -value ${}^{*}$
Blood oxygen saturation (%)	94.27 ± 1.82	98.88 ± 0.99	< 0.001
OCTA quality	5.48 ± 2.03	5.73 ± 1.89	0.577
Foveal thickness (µ)	249.26 ± 29	245.22 ± 32.16	0.191
FAZ area (mm^2^)	0.38 ± 0.14	0.34 ± 0.12	0.035
FAZ area (mm^2^) in males	0.39 ± 0.15	0.36 ± 0.11	0.182
FAZ area (mm^2^) in females	0.39 ± 0.15	0.33 ± 0.11	0.203
FAZ perimeter (mm)	2.46 ± 0.56	2.37 ± 0.46	0.145
Acircularity index	1.13 ± 0.10	1.14 ± 0.05	0.532
SCP VD at fovea (%)	13.5 ± 6.37	14.98 ± 6.33	0.059
SCP VD at fovea (%) in males	12.33 ± 5.04	13.48 ± 3.4	0.234
SCP VD at fovea (%) in females	14.27 ± 7.66	15.82 ± 8.12	0.275
SCP VD at temporal parafoveal quadrant (%)	38.67 ± 4.88	40.56 ± 6.16	0.052
DCP VD at fovea (%)	24.61 ± 6.75	26.59 ± 6.16	0.022
DCP VD at fovea (%) in males	24.53 ± 6.28	26.04 ± 4.33	0.115
DCP VD at fovea (%) in females	24.37 ± 7.84	25.79 ± 6.53	0.199
OCTA, optical coherence tomography angiography; FAZ, foveal avascular zone; SCP, superiorerficial capillary plexus; DCP, deep capillary plexus; VD, vessel density ${}^{*}$ Based on paired t-test			

**Table 2:** Retinal changes in males and females with DMI after normobaric oxygen therapy

**Parameter**	**Male**	**Female**	***P*** -value ${}^{*}$
Pre-O ${}_{2}$ parafoveal thickness	310.27 ± 22.63	310.18 ± 22.95	0.993
Post-O ${}_{2}$ parafoveal thickness	310.87 ± 22.55	309.55 ± 23.51	0.886
*P*-within ${}^{*}$	< 0.001	< 0.001	
Pre-O ${}_{2}$ parafoveal superior hemi thickness	315.2 ± 21.82	311.27 ± 26.42	0.682
Post-O ${}_{2}$ parafoveal superior hemi thickness	315.33 ± 23.74	310.55 ± 26.81	0.635
*P*-within ${}^{*}$	< 0.001	< 0.001	
Pre-O ${}_{2}$ parafoveal inferior hemi thickness	305.27 ± 27.03	308.82 ± 21.22	0.721
Post-O ${}_{2}$ parafoveal inferior hemi thickness	306.27 ± 23.88	308.18 ± 22.1	0.837
*P*-within ${}^{*}$	< 0.001	< 0.001	
Pre-O ${}_{2}$ parafoveal superior thickness	316.33 ± 24.86	314.73 ± 30.05	0.883
Post-O ${}_{2}$ parafoveal superior thickness	317.07 ± 26.9	313.64 ± 31.16	0.766
*P*-within ${}^{*}$	< 0.001	< 0.001	
Pre-O ${}_{2}$ parafoveal inferior thickness	304.33 ± 27.73	307.73 ± 20.86	0.736
Post-O ${}_{2}$ parafoveal inferior thickness	305.33 ± 23.64	307.09 ± 21.81	0.848
*P*-within ${}^{*}$	< 0.001	< 0.001	
Pre-O ${}_{2}$ parafoveal nasal thickness	319.27 ± 22.43	314.36 ± 24.47	0.601
Post-O ${}_{2}$ parafoveal nasal thickness	317.8 ± 26.49	313.64 ± 24.57	0.687
*P*-within ${}^{*}$	< 0.001	< 0.001	
Pre-O ${}_{2}$ parafoveal temporal thickness	300.8 ± 32.11	303.82 ± 23.4	0.794
Post-O ${}_{2}$ parafoveal temporal thickness	302.8 ± 28.96	302.73 ± 23.82	0.995
*P*-within ${}^{*}$	< 0.001	< 0.001	
DMI, diabetic macular ischemia ${}^{*}$ Based on paired t-test			

**Table 3:** Baseline OCTA parameters showing significant correlations with logMAR visual acuity in males and females

**Gender**	**Pre-O ${}_{2}$ parameter**	Correlation coefficient ${}^{*}$	***P*** **-value**
Male	DCP VD whole image	–0.508	0.050
	DCP VD superior hemi	–0.541	0.037
	DCP VD perifovea	–0.529	0.043
	DCP VD perifoveal superior hemi	–0.512	0.051
	DCP VD perifoveal superior quadrant	–0.606	0.017
Female	DCP VD perifoveal inferior quadrant	0.624	0.040
OCTA, optical coherence tomography angiography; logMAR, logarithm of minimum angle of resolution; DCP VD, deep capillary plexus vessel density ${}^{*}$ Pearson correlation			

### Subgroup Gender-based Analysis 

At baseline, males and females were not different in terms of the study parameters. Both genders showed a significant change in the retinal thickness of the parafoveal region. All females showed a significant decrease in the retinal thickness of the parafoveal area (all *P* < 0.001), while in males, all areas except the nasal area showed a significant increase in parafoveal retinal thickness [Table 2]. There was no significant difference regarding changes in perifoveal retinal thickness, SCP-VD, and DVP-VD between males and females. Both genders showed a non-significant decrease in FAZ area and an increase in foveal SCP-VD and DCP-VD [Table 1].

### Correlation Between BCVA and OCTA Parameters

There was no significant correlation between BCVA and baseline FAZ area, perimeter, and none of the foveal parameters in either gender. However, in males, BCVA showed a statistically significant correlation with pre-NBO DCP-VD of the whole image (*r* = –0.508, *P =* 0.050), superior hemi (*r* = –0.541, *P = *0.037), perifovea (*r* = –0.529, *P = *0.043), perifoveal superior hemi (*r* = –0.512, *P = *0.050), and perifoveal superior quadrant (*r* = –0.606, *P = *0.017). None of the pre-NBO SCP-VD parameters significantly correlated with baseline BCVA [Table 3]. Post-NBO parameters with a statistically significant correlation with BCVA included SCP-VD of parafoveal superior hemi (*r* = –0.521, *P = *0.046), parafoveal nasal quadrant (*r* = –0.512, *P = *0.050), perifoveal nasal quadrant (*r* = –0.548, *P = *0.043), and DCP-VD of parafovea (*r* = –0.550, *P = *0.034), parafoveal inferior hemi (*r* = –0.643, *P = *0.010), parafoveal nasal quadrant (*r* = –0.631, *P = *0.012), perifovea (*r* = –0.535, *P = *0.040), perifoveal inferior hemi (*r* = 0.533, *P = *0.041), and perifoveal superior quadrant (*r* = –0.586, *P = *0.022). In females, BCVA showed a significant correlation with pre-NBO DCP-VD of the perifoveal inferior quadrant (*r* = –0.624, *P = *0.040) and post-NBO DCP-VD of the parafoveal superior quadrant (*r* = –0.614, *P = *0.044) [Table 3].

For each parameter, we compared the number of eyes with favorable responses (decrease in retinal thickness or increase in VD) to those without response. We observed a significant difference for FAZ area (*P = *0.059) and foveal DCP-VD (*P = *0.036). FAZ area decreased in 16 eyes (62%), and foveal DCP-VD increased in 18 eyes (69%); however, regression analysis did not show any parameter (such as age, gender, and baseline VA) predicting a favorable response.

##  DISCUSSION

This study extends previous studies on the application of NBO therapy for DMI by demonstrating significant changes in OCTA metrics after 1 hour of systemic oxygen administration. We observed a significant decrease in FAZ area (*P = *0.035) and an increase in foveal DCP-VD (*P = *0.022) but a marginally significant increase in foveal SCP-VD (*P = *0.059). Retinal thickness did not change significantly, as patients with significant retinal edema and CI-DME were excluded from the study. However, there was a significant decrease in parafoveal retinal thickness in females and a significant increase in male patients. These observations align with previous studies reporting improvement in the structure or function of the retina in patients with macular ischemia after hyperoxia.^[13,14][19]^

In recent years, OCTA has been largely used to investigate retinal vessels. OCTA has several advantages over FA: it is a noninvasive method based on motion contrast without the need for injecting a contrast agent. It generates volumetric data of superficial and deep retinal vessels. However, artifacts are more commonly observed with OCTA images than FA. Holmen et al found at least one artifact in 97.3% of images and severe artifacts in 53.5% of scans. In their study, the most common artifacts were shadow (26.9%), defocus (20.9%), and movement (16%).^[20]^ Other disadvantages of OCTA include the limited field of view and the inability to identify leakage. In our study, we removed some eyes due to artifacts in either pre- or post-NBO images.

A few studies have compared OCTA and FA in patients with DMI and have found moderate agreement between DMI grading with OCTA and conventional FA using standard ETDRS protocols. Still, there is no widely accepted system for OCTA-based classification. Cheung et al classified DMI into three distinct clinical phenotypes using OCTA: generalized DMI, predominant-DCP ischemia, and predominant-SCP ischemia.^[22]^ Recent studies have shown that DCP-DMI is more prevalent and correlates more strongly with functional deficits than SCP-DMI.^[23,24]^ The present study showed a significant increase in foveal DCP-VD (P = 0.022).

Retina is among the most metabolically active tissues in the body with an oxygen consumption rate of 13 mL/100 g/min, which makes it sensitive to changes in the oxygen supply.^[25]^ In diabetes, biochemical mechanisms induced by hyperglycemia result in pericyte loss, thickening of the basement membrane, and endothelial cell damage, contributing to the impairment of the blood–retinal barrier. These, in turn, will increase vascular permeability and retinal edema on the one hand and vascular occlusion and retinal ischemia on the other. The final outcome of ischemia would be the upregulation of VEGF, neovascularization, and PDR.^[26,27]^ Although the retinal vascular response to a hyperoxic state differs in patients with diabetes compared to healthy individuals,^[28]^ hyperoxia may favorably affect DMI by increasing the amount of oxygen dissolved in the plasma and, thereby, increasing the oxygen gradient between the blood and the retina to transfer more oxygen to the tissue. Additionally, oxygen-induced vasoconstriction decreases retinal edema and decreases the distance for oxygen to diffuse. As a result, it ameliorates ischemia and improves retinal function. Furthermore, improving ischemia reduces VEGF production. We observed a decrease in FAZ and an increase in DCP after short-term NBO therapy, although the exact mechanisms behind our findings are unknown and beyond the scope of this study.

In a pilot study on 20 eyes with severe DMI, after 1 hour of 100% systemic oxygen therapy by face mask at a flow of 10 L/min, significant improvement occurred in retinal function (in terms of ERG b-wave amplitude) and retinal structure (in terms of central macular thickness).^[13]^ In a randomized controlled trial on 90 eyes of 90 patients with DMI, the patients were randomized to oxygen therapy, enalapril, and control groups. In the oxygen therapy arm, the patients received 100% oxygen at a flow rate of 10 L/min by face mask. The regimen started at 1 hour twice daily for the first month, once daily for the second month, and every other day for the third month, then followed for three additional months. The primary outcomes, including BCVA, FAZ area measured by FA, and CMT measured by OCT, were evaluated at baseline, month three, and month six. Significant improvement in BCVA, FAZ area, and CMT occurred in the oxygen cohort, compared to deterioration in the control and enalapril groups.^[14]^

The present study, using OCTA, adds to the existing data on the potential benefits of oxygen therapy for DMI. Following 1 hour of NBO therapy as described earlier, FAZ area decreased (*P = *0.035) and foveal DCP-VD increased significantly (*P =* 0.022). The increase in foveal SCP-VD was not significant (*P = *0.059), which could be attributed to the small sample size. In this series of patients, a favorable response, as defined earlier, was observed in about two-thirds of the patients. FAZ decreased in 16 eyes (62%), and foveal DCP-VD increased in 18 eyes (69%), but regression analysis did not indicate any parameter predicting a beneficial change.

In a study evaluating retinal vascular response to hyperoxia, healthy individuals showed a reduction in parafoveal DCP-VD due to autoregulatory mechanisms; however, such a reduction was not observed in diabetic patients without retinopathy.^[28]^ Impaired autoregulation might play a role in increased VD, which was observed in patients with DMI in our study and might ultimately benefit them. Since DCP-VD has been correlated with BCVA, the significant increase in DCP after a short period of hyperoxia might be considered clinically relevant.

Several studies have shown the effects of supplemental oxygen on retinal function or structure in diabetes with^[10,11,12][29][30]^ or without retinopathy^[28]^ and other retinal ischemic conditions^[19,31]^ or retinal vascular occlusions.^[32,33,34]^ We observed no significant change in retinal thickness, as patients with retinal edema and CI-DME were excluded from the study. However, intracellular edema may increase macular thickness, and improvement of ischemia may lead to a decrease in macular thickness by reducing intracellular edema. Previous studies have shown that females have greater FAZ than males.^[16,17,18]^ Therefore, we performed a subgroup analysis and observed no baseline difference in retinal thickness between the two genders. However, after NBO therapy, females showed a significant decrease in the thickness of all parafoveal sectors (all *P* < 0.001), while in males, all parafoveal sectors except the nasal one showed a significant increase in retinal thickness [Table 2]. These opposite changes counteracting each other resulted in an overall non-significant change in retinal thickness. Although the thickness changes are small and could be clinically not significant, the opposite direction of changes in males and females might be clinically relevant. Future studies with DME should address these potential gender differences in response to NBO therapy.

In our study, there was no significant correlation between foveal parameters and BCVA. However, subgroup analysis for gender showed a significant negative correlation between BCVA and pre-NBO DCP-VD of the whole image, superior hemi, perifovea, perifoveal superior hemi, and perifoveal superior quadrant in males. None of the pre-NBO SCP-VD parameters showed a significant correlation with baseline BCVA. Statistically significant correlations found between BCVA and post-NBO parameters might not be clinically relevant. In females, the only pre-NBO parameter showing a significant correlation with BCVA was DCP-VD of the perifoveal inferior quadrant [Table 3].

Our findings align with previous studies showing the correlation between FAZ area and VD in the deep vascular plexus with visual acuity.^[1,35,36]^ Sim et al observed a reduction in visual acuity only in moderate to severe ETDRS-DMI grades of ischemia with FAZ areas of 0.32 and 0.78 mm^2^ and BCVA of 0.5 and 0.6, respectively.^[1]^ In our study, FAZ area and BCVA were 0.38 ± 0.14 and 0.41 ± 0.27 mm^2^, respectively. However, we observed significant correlations between BCVA and DCP-VD, but not with the FAZ area. In a study on diabetic patients with DME, Kim et al reported a significantly lower DCP-VD and a significant correlation between SCP-VD and FAZ area with BCVA.^[37]^ Even in eyes undergoing vitrectomy for DME, preoperative macular ischemia with enlarged FAZ was correlated with worse postoperative BCVA.^[38]^

The limitations of the present study include the small sample size and a short-term follow-up. Additionally, we did not measure BCVA after oxygen therapy. We did not evaluate peripheral retinal ischemia and ischemic index either and considered the increase in FAZ size and decrease in VD as indirect indicators of macular ischemia. We also used the ETDRS classification for DMI, which is based on FA and not OCTA. Patients with COPD were excluded, and no adverse effects were observed following oxygen therapy. We used the flow rate and duration reported in previous studies;^[13,14]^ however, future studies may suggest different treatment regimens.

In summary, our study using OCTA showed that hyperoxia might have beneficial effects in DMI by decreasing FAZ and increasing foveal DCP-VD. However, future studies with a larger sample size and longer follow-up are needed to evaluate the functional and structural effects of NBO therapy and its related mechanisms in DMI and the optimal flow and duration of the treatment.

##  Financial Support and Sponsorship

None.

##  Conflicts of Interest

None.
